# Permissiveness of different TMEM154 genotype cell lines to different SRLV genotypes/subtypes

**DOI:** 10.1128/jvi.00961-25

**Published:** 2025-08-22

**Authors:** Barbara Colitti, Daniele Avanzato, Riccardo Moretti, Irati Moncayola, Margherita Profiti, Stefania Chessa, Paola Sacchi, Sergio Rosati, Ramses Reina

**Affiliations:** 1 Department of Veterinary Sciences, University of Turin 9314, Turin, Italy; 2 Instituto de Agrobiotecnología (CSIC-Gobierno de Navarra) 98696 , Mutilva Baja, Navarra, Spain; Icahn School of Medicine at Mount Sinai, New York, New York, USA

**Keywords:** SRLV, lentiviruses, genotypes, virus pseudotypes, genetic selection, genetic resistance

## Abstract

Small ruminant lentiviruses (SRLVs) are a heterogeneous group of viruses that infect goats and sheep worldwide, for which no cures or effective vaccines are available. Control measures have been based on early diagnosis and accurate management with limited success due to re-emergence of seropositivity and disease in controlled flocks. Association between the genetic variation in the ovine transmembrane 154 (*TMEM154*) gene and susceptibility to certain viral subtypes was previously demonstrated, representing a possible control strategy to reduce infection prevalence in sheep. Thus, the identification of the resistance spectrum of E35K SNP within *TMEM154* genotypes toward SRLV subtypes becomes crucial. In this study, 10 skin fibroblastic cell lines, from animals encoding three genotypes of the *TMEM154* (E35K) SNP, were infected with 8 SRLV viral strains and tested for the presence of retrotranscriptase (RT) activity and cytopathic effect. Additionally, SRLV entry into cells was evaluated using viral pseudotypes. Two out of the eight viral strains showed fewer syncytia, reduced cell fusion degree, and lower RT activity in the supernatant of homozygous KK cell lines, suggesting a resistant pattern in these cells. The entry assay revealed a statistically significant difference between the three cell lines in seven of the eight strains, suggesting an entry blockade as the primary restriction factor in KK cells. These findings support the potential use of the *TMEM154* gene as a marker for the genetic selection of resistant animals to specific SRLV subtypes. Moreover, the *ex vivo* approach was validated as a valuable tool for studying resistance patterns against different SRLV strains.

**IMPORTANCE:**

Small ruminant lentiviruses are worldwide spread pathogens that impact animal health and result in severe economic losses. Considering the high genetic and antigenic variability of these viruses and the absence of an effective cure or vaccine, the genetic selection of resistant animals based on the *TMEM154* gene represents an interesting opportunity to control the infection. Thus, this study aimed to investigate further the host-pathogen interaction considering the association between the animal genotype for the suggested protective mutation and the infecting virus genotype using an *in vitro* model. The study confirmed *TMEM154* genetic variation as a helpful predictive factor for SRLV susceptibility by particular SRLV strains including the highly pathogenic B1 subtype, while susceptibility to some A and the B2 subtypes was not affected by the *TMEM154* gene. Once more, it highlighted the importance of monitoring circulating viral variants for the effective control of SRLV infection through genetic selection programs.

## INTRODUCTION

Small ruminant lentiviruses (SRLVs) are a heterogeneous group of viruses, belonging to the genus *Lentivirus*, family *Retroviridae*, that cause chronic multisystemic infections clinically characterized by pneumonia, wasting, arthritis, encephalitis, paralysis, and indurative mastitis that end up in important economic losses in the small ruminant industry due to increased mortality and reduced milk production (1–3).

Initially distinct viral species, caprine arthritis encephalitis virus (CAEV) affecting goats and Maedi-Visna virus (MVV) affecting sheep were later reassigned by phylogenetic analyzes as closely related lentiviruses grouped together as SRLV.

Several SRLV variants have been identified (4–7), leading to a classification into four genotypes. Genotype A is the most heterogeneous group, which contains classical maedi-visna viruses and other SRLV variants, including some that infect both sheep and goats. Genotype B contains classical CAEV strains, mainly isolated in goats along with other strains (i.e., B2 and B3) isolated in sheep. Genotypes A and B are worldwide spread (2). Regarding the remaining poorly diffused genotypes, genotype C affects both sheep and goats and is restricted to Norway and Sweden (8–10), while genotype E to date only occurs in goats and is restricted to Italy (2).

Although control strategies have been implemented in many countries (11–17), the insidious features of the disease and the high variability of the infectious agent have led to a worldwide distribution of SRLV with high prevalences, except for Iceland, New Zealand, and Australia where a MVV free status was gained after an intense eradication program. In the absence of cures or effective vaccines, control measures are based on early diagnosis, elimination of seropositive animals, and accurate management (7, 18). However, delayed seroconversion, fluctuating antibodies, and antigenic heterogeneity hinder identification of infected animals (7).

The role of breed on SRLV seroprevalence has been highlighted in the last decades by some research studies suggesting a host genetic influence on infection susceptibility (19–21).

An association between the genetic variation in the ovine transmembrane 154 (TMEM154) protein coding gene and infection susceptibility to certain SRLV subtypes was demonstrated with a genome-wide association study (GWAS) approach (22). Additional sheep genes, such as MHC, CCR5, TLRs, APOBEC3, and ZNF389, have been linked to SRLV infection (23).

Several haplotypes have been identified based on sequence variation in the TMEM154 coding region. Among them, three haplotypes have been well characterized in several studies (20, 24–28). Two *TMEM154* haplotypes (2 and 3, ancestral), carrying a nucleotide (G) encoding for glutamic acid (E) at position 35, were associated with infection, while haplotype 1 encodes a lysine (K) at position 35 and has been associated with resistance (20).

The reduced susceptibility to SRLV infection of certain *TMEM154* polymorphisms was confirmed in several studies that highlighted how both, host and SRLV genetic subgroups, strongly affect the risk of infection in sheep, especially considering the high evolution rate of these viruses (22, 25, 26, 29–32).

Thus, the selection based on *TMEM154* polymorphisms represents a possible control tool for reducing the prevalence of infection in sheep. However, considering the biological complexity of SRLV, the host/pathogen interaction warrants further exploration.

The aim of the present study was to identify the *in vitro* susceptibility/resistance spectrum of different *TMEM154* genotypes toward different SRLV subtypes in sheep. To achieve this aim, 10 fibroblastic cell lines, harboring three genotypes of the *TMEM154* gene (4 KK, 3 EK, and 3 EE), were infected with 8 SRLV viral strains and tested for the presence of retrotranscriptase (RT) activity and cytopathic effect. Moreover, an entry assay, using viral pseudotypes, was performed to evaluate if resistance was ascribable to a differential ability of SRLV in entering the cell or to post-entry restriction.

## MATERIALS AND METHODS

### Cells and viruses

Ten ovine skin fibroblast cell lines were obtained by skin biopsy as a diagnostic work-up, from SRLV-seronegative animals identified as carriers of the three different *TMEM154* genotypes by ligase detection reaction (LDR) (33) in a previous work (32).

Briefly, skin punch biopsies were immediately put in transport medium (Dulbecco’s modified Eagle’s medium [DMEM] [Sigma]) supplemented with 2% L-Glutamine (Sigma), 0,1% Gentamycin, and 2% antibiotic/antimycotic solution (Sigma) and immediately transferred to the laboratory for further processing. The skin samples were chopped into small pieces using a sterile disposable scalpel and forceps and incubated for 4 hours at 37°C, 5% CO_2_ in a humidified incubator with transport medium. Then tissues were transferred in 25 cm^2^ flasks with the same medium supplemented with 10% fetal bovine serum (FBS). The cells were propagated for three passages and then stored in liquid nitrogen.

Embryonic kidney 293T cells and ovine fibroblasts were maintained in DMEM medium supplemented with 5% (HEK293T) or 10% (fibroblasts) FBS (GIBCO), 2% L-Glutamine (Sigma), and 1% antibiotic/antimycotic solution (Sigma) and grown in a humidified atmosphere of 5% CO_2_ at 37°C.

Eight viral strains were used in this study for *in vitro* infection and RT activity analysis, four strains belonging to genotype A (viz., WLC-1 and EV-1 [A1 subtypes], It561 [A3 subtype], and VDA [A8 subtype]) and four strains belonging to genotype B (viz., TO1/89 [B1 subtype], It2038 and Ov496 [B2 subtype], and Volterra [B3 subtype]), kindly provided by Maurizio Mazzei (University of Pisa, Italy) (Table 1). The strains belonged to the viral collection of the laboratory, where each strain and the assigned subtype had been previously confirmed by Sanger sequencing or next Generation sequencing (4).

**TABLE 1:** Names and subtypes of SRLV viral strains used in the present study

Strain	Genotype	Subtype
WLC1	A	A1
EV1	A	A1
K1514	A	A1
85/34	A	A2
It561	A	A3
Sp258	A	NA
CAEV TO1/89	B	B1
CAEV63	B	B1
It2038	B	B2
Ov496	B	B2
Volterra	B	B3
VDA	A	A8
Seui	E	E2

Viral infectivity and the correct multiplicity of infection (MOI) of each strain were determined on subconfluent permissive ovine fetal lung cells in 96-well tissue culture plates, by end-point dilution method after 7 days of infection. For each strain, eight replicates of serial 10-fold dilutions (from 10^−1^ to 10^−7^) of the viral stock were tested together with two negative controls. The monolayers were then stained with May-Grünwald Giemsa and examined for the presence of syncytia. Viral titers were calculated using the Reed-Muench method (34) and expressed as tissue culture infectious dose (TCID_50_) per milliliter of supernatant.

### *In vitro* infection and RT activity

Ten fibroblastic cell lines, encoding the three different *TMEM154* genotypes (3 EE, 4 KK, and 3 EK), were infected with the eight viral strains at MOI of 0.1–1. An uninfected cell line was used as a negative control.

The day before infection, cells were seeded in 24-well tissue culture plates (1.5 × 10^5^ cells/well) using the complete medium and then incubated overnight at 37°C in a modified, 5% CO_2_ atmosphere. The culture medium was replaced every 4–7 days. The supernatant was collected at days 0, 2, 5, 8, 12, 15, and 19 post-infection (p.i.) and tested for the presence of RT activity. At day 19, once fixed and stained with May-Grünwald Giemsa, the cells were analyzed for the presence of syncytia. The number and morphology of syncytia were recorded.

RT activity was evaluated through SG-PERT SYBR Green assay (35, 36) as previously described.

Briefly, virions contained in 10 µL of supernatant were lysed with 2× lysis buffer (0.25% Triton X-100, 50 mM KCl, 100 mM Tris-HCl pH 7.4 and 40% glycerol, and 2% of RNAse inhibitor (RiboLock, ThermoFisher Scientific, Waltham, MA, USA) right before usage and incubated at room temperature for 10 min.

The viral lysate was diluted 1:5 in a sample dilution buffer (5 mM (NH_4_)_2_SO_4_, 20 mM KCl, 20 mM Tris-HCl pH 8.3) and added to a master mix containing RNA from bacteriophage MS2 (Sigma-Aldrich, St. Louis, MO, USA), RNAase inhibitors (RiboLock, ThermoFisher Scientific, Waltham, MA, USA), MS2 primers, and SYBR Green I for retrotranscription and quantification following the protocol described by Vermeire (35) in a CFX OPUS96 Thermocycler (Biorad). Each sample was tested in triplicate, and a standard curve, obtained with dilutions of titrated HIV-1 and SRLV WLC-1 stocks, was constructed and performed in each analysis for quantification.

Data were analyzed using R software (v 3.4.2) (37). The correlation between *TMEM154* E35K genotype and susceptibility to infection with different SRLV subtypes was evaluated considering the RT activity quantification cycle (Cq) values recorded for the three *TMEM154* genotypes for each viral subtype, at each time of infection using the Wilcoxon-Mann-Whitney test. Results with *P*-values lower than 0.05 were deemed as statistically significant.

### Production of pseudotyped viruses

Pseudotype production requires the availability of a cloned envelope, a feature that could not be achieved in all the eight viruses used for *in vitro* infection and RT activity analysis. Therefore, we obtained full-length *env* genes of homologous strains from the same subtypes, namely, K1514 (A1 subtype) (38), 85/34 (A2 subtype) (38), CAEV63 (B1 subtype) (39), Seui (E2 subtype) (40–42), and Sp697 (A3 subtype belonging to an outbreak of neurological disease) (42, 43), Sp258 (A subtype belonging to an outbreak of pulmonary disease) (44), TO1/89 (B1 subtype) (45) and Volterra (B3 subtype) (46) strains were used to generate env-expressing constructs using pCMV plasmid, a low copy number expression vector, and CAEV-AP backbone (including a thermostable human placental alkaline phosphatase [HuPAP) as reporter gene).

The plasmids and pCMVk1514, pCMV85/34, and pCMVCAEV63 were kindly provided by Prof. Hotzel (39), pCMVSeui, pCMV697, pCMV 258, pCMVTO1/89, and pCMVVolterra were produced as previously reported (39).

Briefly, fetal caprine lung (PFO) and fetal ovine lung (PFO) cell lines were infected with TO1/89 and Volterra viruses, respectively. Viral genomes were isolated from the extracellular medium to amplify the full coding sequence of envelope gene with AllTaq Master Mix kit (QIAGEN) using specific forward and reverse primers carrying BamHI and EcoRI restriction site respectively (TO1/89 forward: 5′- TTGGATCCACCATGGATGCCGGGGCAAAATACATAG −3′; TO1/89 reverse: 5′- TTGAATTCTCACAGTCCACCCTTTCTTTTTC −3′; Volterra forward: 5′- TTGGATCCACCATGGATTGCGGGGCTCGTGAAATAC −3′; Volterra reverse: 5′- TTGAATTCTCAGTCCTCTTTCTCCCAGCTCTC −3′). Samples (800 ng of genomic DNA) were subjected to 40 cycles of a denaturing step at 93°C for 31 min, an annealing step at 61°C for 30 sec, and an extension step at 68°C for 2 min and 45 sec. PCR products were then visualized by agarose gel electrophoresis, purified with S.N.A.P. UV-Free Gel Purification Kit (Invitrogen), and subcloned into the low copy number pCMV plasmid after digestion by restriction enzymes *BamHI* and *EcoRI*.

### Entry assay

HEK 293 T cells were used to generate pseudotyped virions by co-transfection of the pCAEV-AP plasmid with envelope proteins from the eight different SRLV strains as previously reported (47). Briefly, HEK 293 T cells were plated in a 6-well multiwell plate (2.5*10^5^ cells for each well) and transfected the day after with Jet Prime Transfection Reagent (PolyPlus) with 2 µg of total DNA (1.8 µg of pCMV CAEV-AP and 0.2 µg of envelope plasmid) with a ratio 1:2 DNA:JetPrime reagent. Supernatants containing the pseudotyped viruses were collected 48 h post-transfection and clarified at 5,000 g for 20 min at 4°C and stored at −80°C.

Ovine fibroblast cell lines with different *TMEM154* genotypes (3 KK, 2 EK, and 3 EE) were seeded in a 24-well multiwell plate (1*10^5^ cells for each well) for the entry assay experiment. The day after cells were infected with pseudotyped virions at different dilutions for 72 h. Cells were then fixed with glutaraldehyde 0.5% (Sigma), and the endogenous alkaline phosphatase was inactivated by heating (65°C for 1 h in PBS). Cells were rinsed once with AP buffer (50 mM MgCl2, 100 mM NaCl, 100 mM Tris pH 8.5) and incubated overnight in the dark at room temperature with BCIP/NBT Liquid Substrate System (Thermo Scientific). The focus-forming units per ml (FFU/mL) were evaluated by counting the stained cells with an inverted optical microscope. Five independent experiments with at least a technical duplicate were performed. The data were presented as mean ± SD. Statistical significance was analyzed by the Kruskal-Wallis test with Dunn’s multiple comparison test with Bonferroni correction in R software (v 3.4.2) (37). *P* < 0.05 was considered statistically significant.

## RESULTS

Ten fibroblastic cell lines have been obtained from animals previously selected as SRLV negative and identified, through LDR assay, as carriers of different genotypes of the gene *TMEM154*, namely, three homozygous EE (GG), three heterozygous EK (GA), and four homozygous KK (AA). Cell lines were propagated for two passages, checked again to confirm the SNP E35K, and infected with eight different SRLV subtypes at MOI of 0.1–1, and monitored for 19 days for the appearance of cytopathic effect or RT activity.

**Fig 1:**
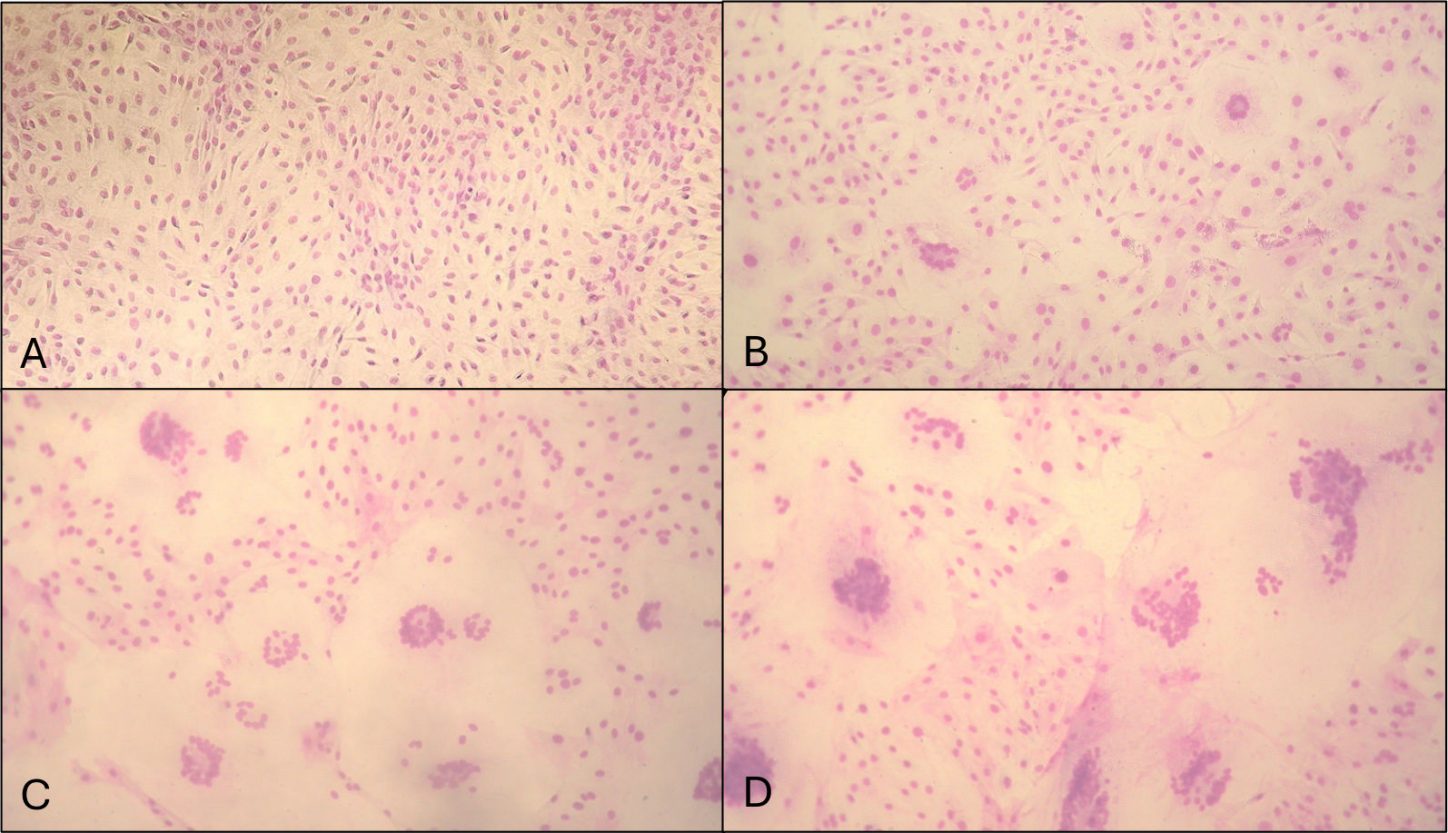
Fibroblastic cell monolayers infected with different SRLV strains showing CPE. (**A**) Intact monolayer. (**B, C, and D**) Monolayer showing small, medium, and big syncytia, respectively.

At day 19 p.i., the cytopathic effect in each cell culture was classified considering a score based on the number and size of syncytia (Table 2 and Fig. 1).

**TABLE 2:** Presence and type of syncytia in ovine fibroblastic cell monolayers, carrying different SNP E35K genotypes, infected with eight SRLV viral subtypes at day 19 p.i.^*a*^

SRLV strain	Cytopathic effect at 19 p.i. for cell lines with SNP E35K genotype:									
	KK	KK	KK	KK	EK	EK	EK	EE	EE	EE
TO1/89	--	--	S+	--	S++	S++	S++	S+	S++	S++
Ov496	S++	S+	S+	L	L	L	S++	S++	S++	S++
It2038	S+++	S+++	S+++	L	L	S+++	S+++	S+++	S+++	S+++
Volterra	S+++	S+++	S+++	S+++	L	L	S+++	S+++	S++	S+++
WLC-1	L/S	L/S	L/S	L/S	L/S	L/S	L/S	L/S	L/S	L/S
EV-1	L/S	L/S	L/S	L/S	L/S	L/S	L/S	L/S	L/S	L/S
VDA	--	--	--	--	--	--	--	--	--	--
It561	--	--	--	--	--	--	--	S+	S+	S+

^*a*^ Type of syncytia were reported as follows: (--) no cytopathic effect; (S+) small syncytia *n* = 0-5/High power field (HPF) (10×); (S++) small-medium syncytia, *n* = 2-10/HPF; (S+++) small, medium and big syncytia, *n* = 5-20/HPF (10×); (L) monolayer with extensive lysis; (L/S) monolayer with lysis and syncytia.

**Fig 2:**
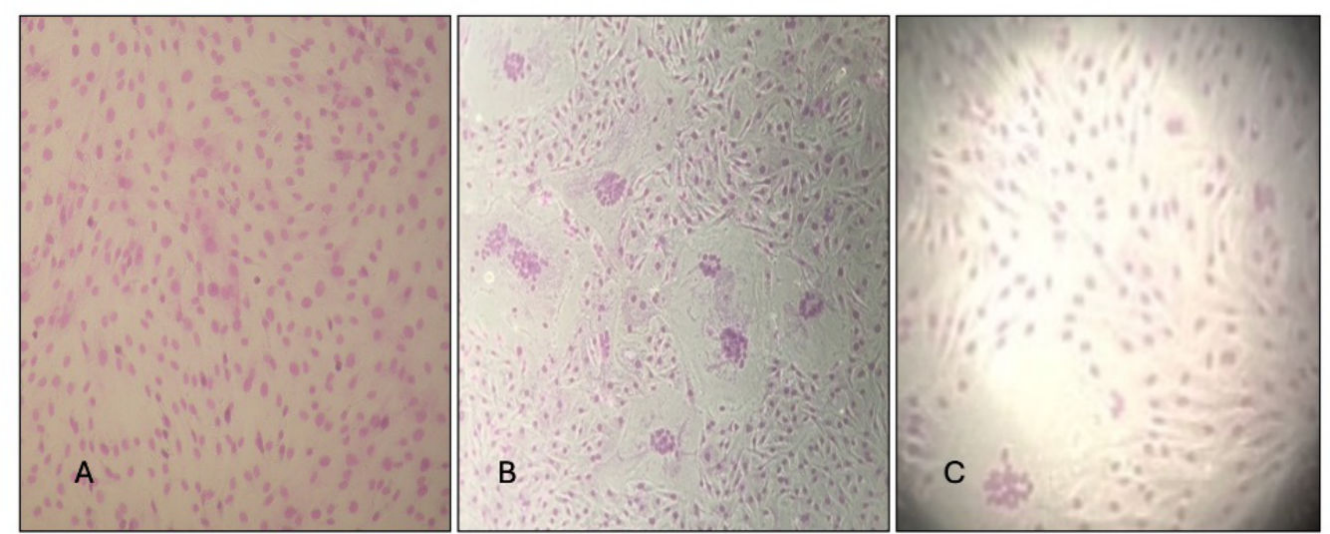
Strain TO1/89, B1 subtype. CPE in cell lines homozygous for the A allele (KK) (**A**) and heterozygous or homozygous for G allele (EK, EE) (**B**). Two small syncytia in one KK cell line (**C**).

At day 19 p.i., the replication of strain TO1/89 (subtype B1) was highlighted in fibroblastic cell lines homozygous and heterozygous for G allele (EK, EE) but not in lines homozygous for A allele (KK), except for a cell line in which two small syncytia were visible (Fig. 2).

**Fig 3:**
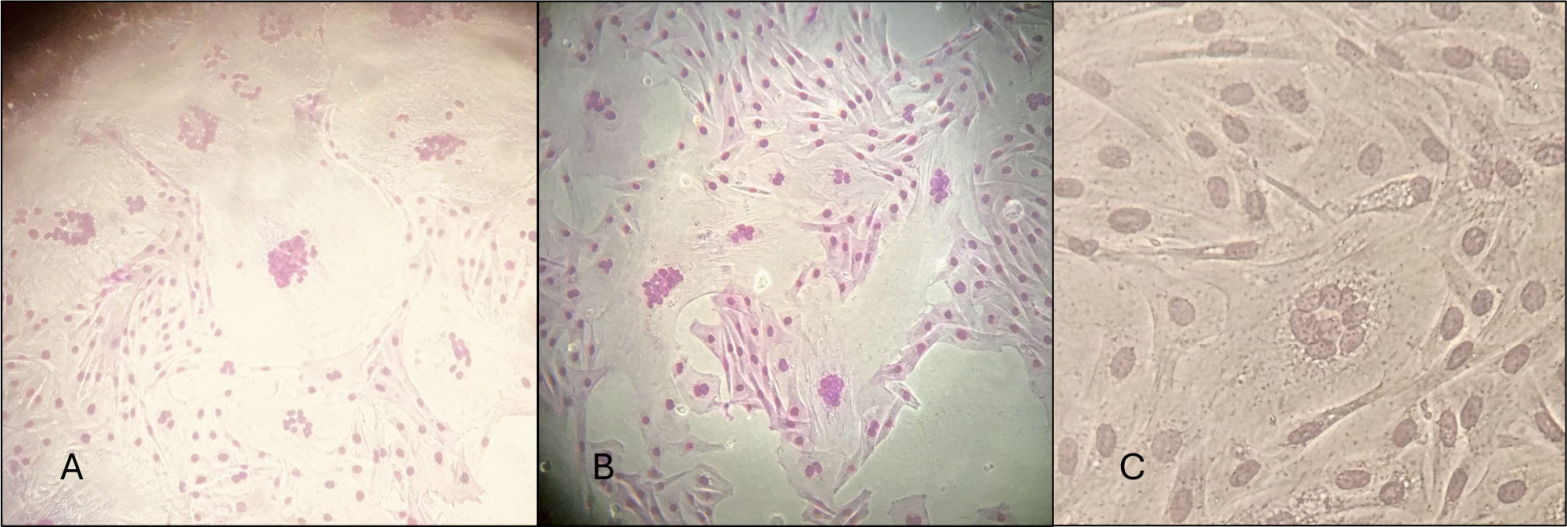
Strain Ov496, B2 subtype. CPE in cell lines homozygous (EE) (**A**) and heterozygous for G allele (EK) (**B**). A small syncytium in one cell line homozygous for A cell line (KK) (**C**).

Regarding the strain Ov496 (subtype B2), all the cell lines supported the replication of the virus with the appearance of syncytia of small and medium size in EE and EK cell lines and a low number of small syncytia in KK cell lines (Fig. 3).

**Fig 4:**
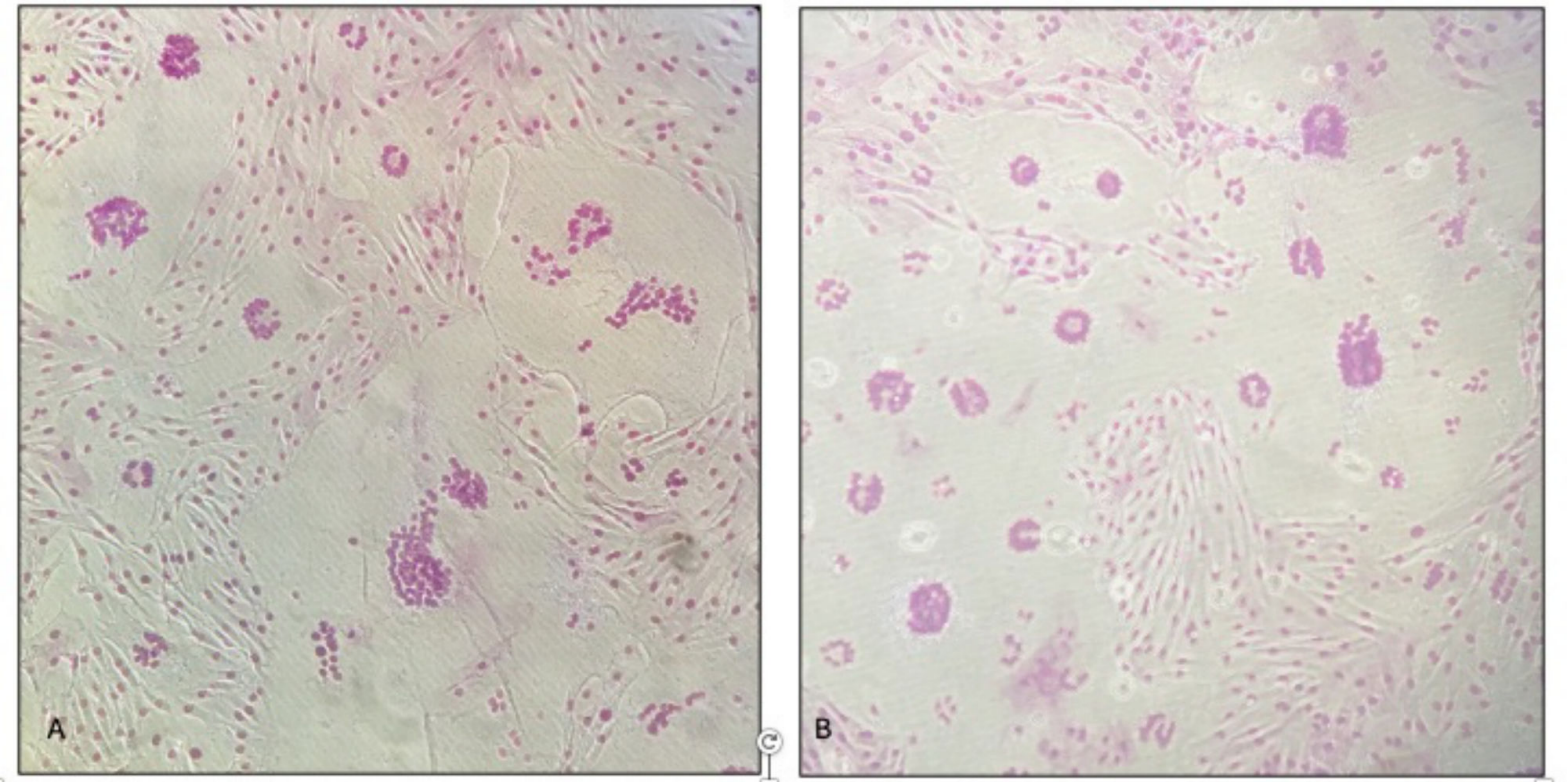
Strain It2038, B2 subtype. CPE in cell lines homozygous and heterozygous for G allele (EE, EK) (**A**) and homozygous for A allele (KK) (**B**).

**Fig 5:**
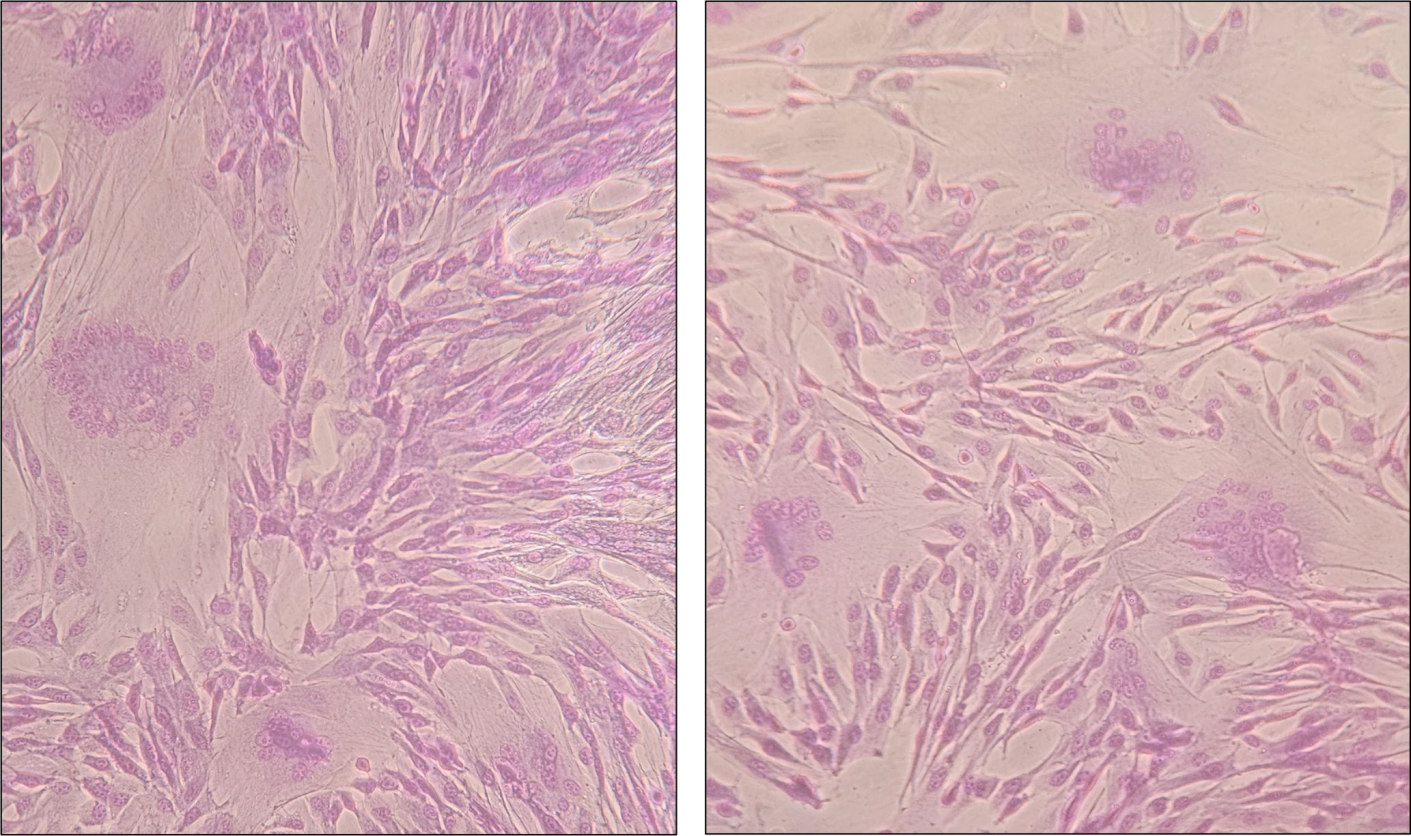
Strain Volterra, B3 subtype. CPE in cell lines homozygous and heterozygous for G allele (EE) (**A**) and homozygous for A allele (KK) (**B**).

The strains It2038 (subtype B2) and Volterra (subtype B3) caused different levels of cell fusion in all cell lines, with small-/medium-sized syncytia in monolayers infected with the previous and medium-/big-sized syncytia with the ones infected with the latter (Fig. 4 and 5).

**Fig 6:**
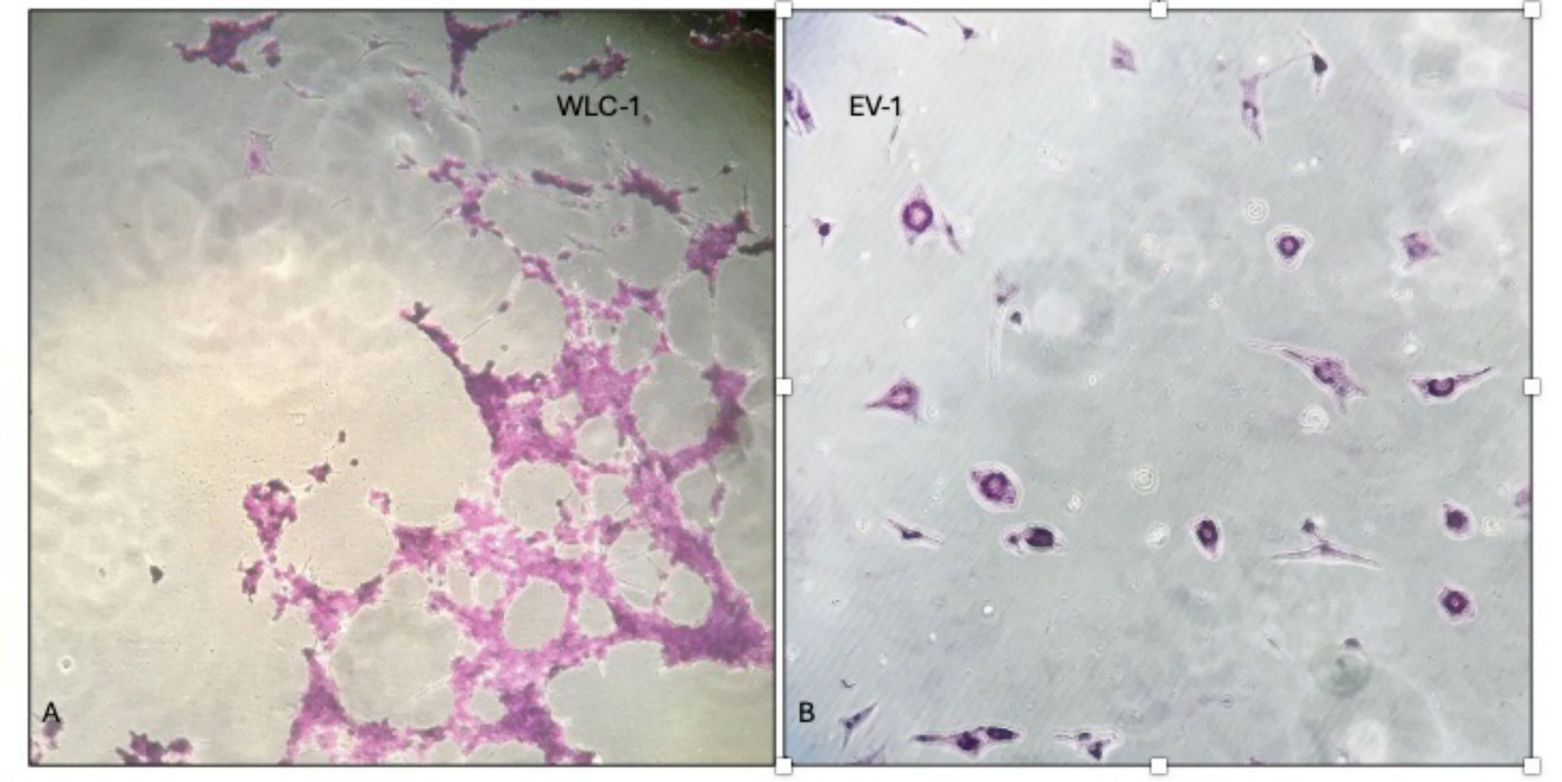
Strain WLC1 and EV1, A1 subtype. CPE in all tested cell lines (homozygous and heterozygous for G allele (EE, EK) and homozygous for A allele (KK)) (A, WLC-1 strainl B, EV-1 strain).

Regarding the A subtype, no cell fusion was recorded for the VDA subtype, while EV1 and WLC1, A1 subtypes, showed extensive cell lysis as well as small syncytia regardless of the cell lines (Fig. 6).

**Fig 7:**
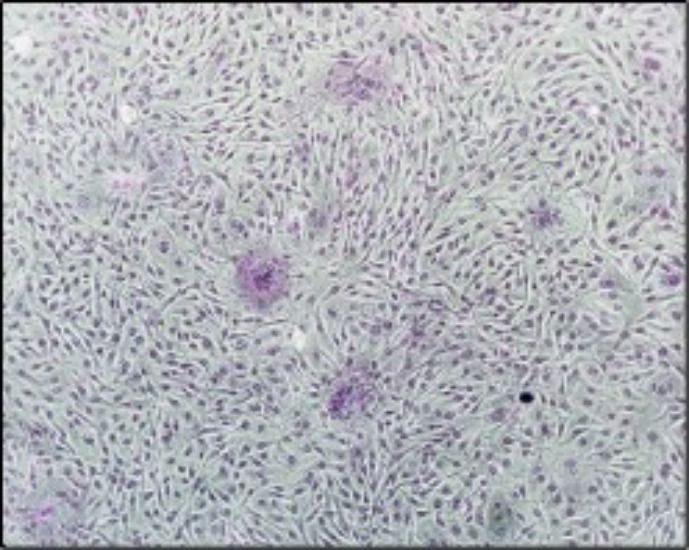
Strain It 561, A3 subtype. A few small syncytia in cell line homozygous for G allele (EE).

Finally, the monolayers infected with It561 strain (subtype A3) did not show any cytopathic effect except for a few small syncytia in the GG (EE) homozygous cell lines (Fig. 7).

**Fig 8:**
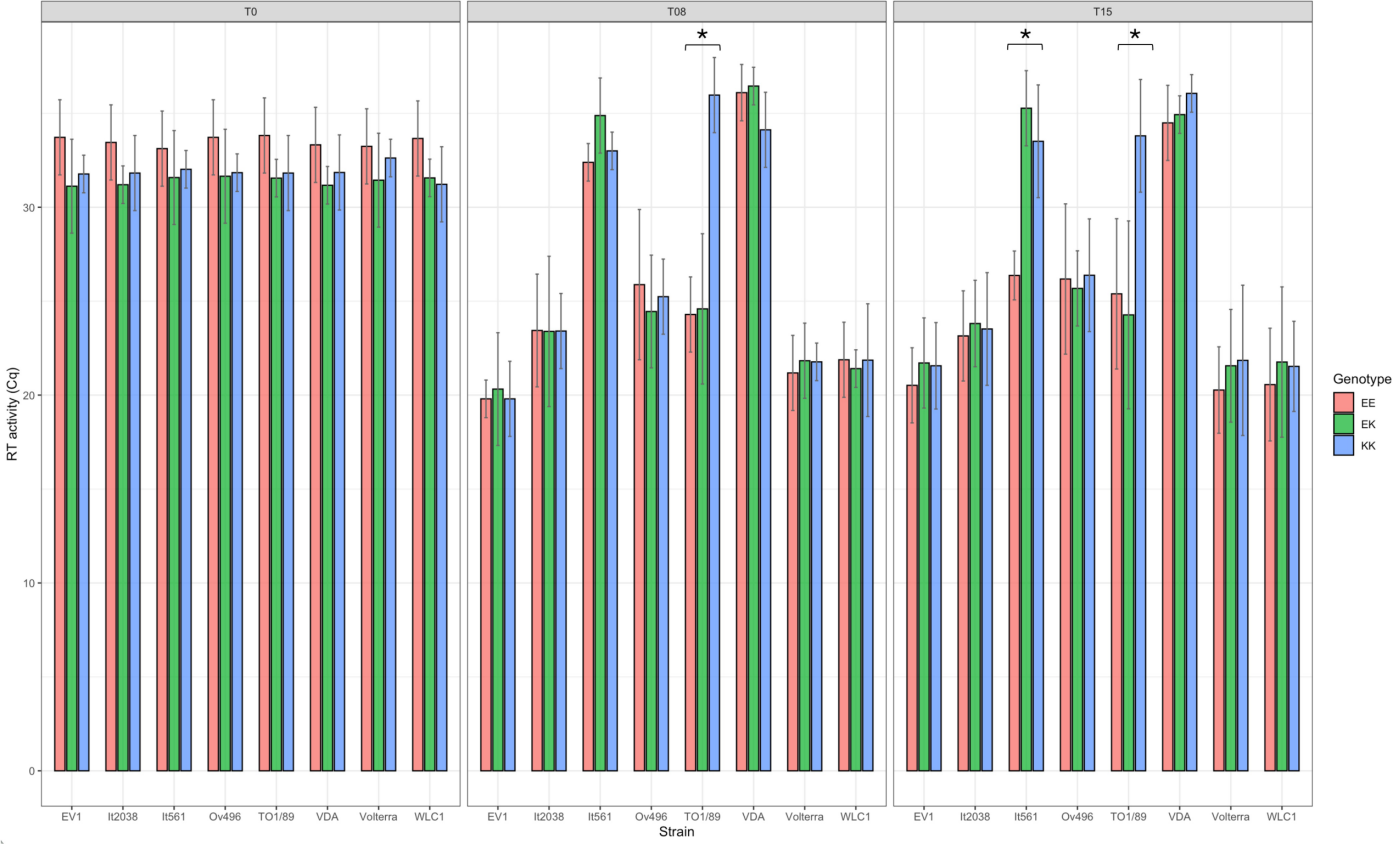
Barplot showing the results of average RT-activity values (expressed as Cq) obtained for three repeated measurements in SG-PERT assay at time 0, 8, and 15 p.i. in clarified supernatants belonging to three different cell line genotypes for *TMEM154* gene and infected with eight different SRLV subtypes. The cutoff for viral replication was assessed at 30 Cq. The error bars show the standard deviation in three replicates. Asterisks denote statistical significance (*P < 0.05; **P < 0.001).

**Fig 9:**
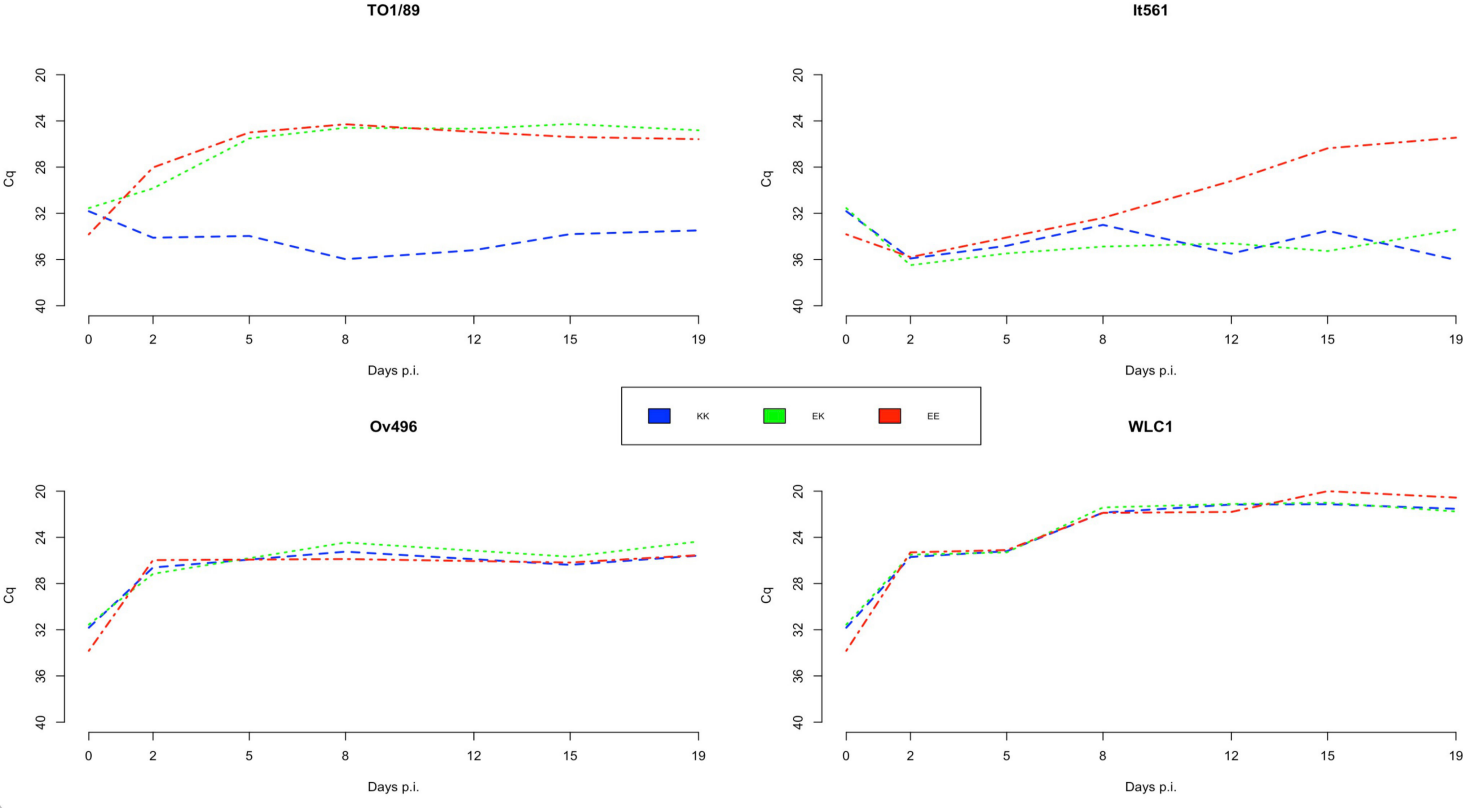
Quantitation of SRLV RT activity by the SG-PERT assay. The plot shows the average of Cq values determined in triplicate, from time T0 to time T19 p.i., in cell lines with different genotypes for the SNP E35K of *TMEM154* locus.

The reverse-transcriptase activity was measured in order to evaluate the virus production in each cell line supernatant at 0, 2, 5, 8, 12, 15, and 19 p.i. Table 3 shows the results obtained on days 0, 8, and 15 p.i. (T0, T8, T15). At T8, SRLV strains WLC1, EV1 (genotype A) and Ov496, and It2038 (genotype B) showed an increase in RT activity in every cell line (Fig. 8 and 9). In particular, an average Cq value of 21.7 and 21.3 was retrieved for WLC1 strain at T8 and T15, respectively, and 19.9 and 21.3 for EV1 strain.

**TABLE 3:** Average RT activity results (expressed as Cq) obtained in the cell lines infected with 8 SRLV strains at time 0, 8, and 15 p.i

Strain	Cq value in cell line haplotype								
	T0			T8			T15		
	KK	EK	EE	KK	EK	EE	KK	EK	EE
Genotype B									
TO1/89	31.82	31.55	33.82	35.97	24.59	24.29	33.8	24.27	25.39
Sp496	31.84	31.65	33.72	25.24	24.45	25.88	26.38	25.68	26.18
It2038	31.82	31.2	33.45	23.41	23.39	23.44	23.52	23.81	23.15
Volterra	32.62	31.44	33.24	21.77	21.83	21.18	21.85	21.56	20.27
Genotype A									
WLC1	31.22	31.56	33.66	21.86	21.41	21.88	21.53	21.76	20.56
EV1	31.77	31.12	33.72	19.8	20.32	19.8	21.56	21.71	20.52
VDA	31.85	31.17	33.32	34.12	36.45	36.1	36.06	34.93	34.49
It561	32.02	31.58	33.12	33	34.88	32.39	33.51	35.27	26.37

The TO1/89 strain replicated only in EK and EE genotypes cell lines, with an average Cq of 24.4 at T8 and 25 at T15 p.i. (Fig. 9), while for the It561 strain, an increase in RT activity was assessed only at T15 and limited to EE cell lines with an average Cq value of 26.5 (Fig. 9).

**Fig 10:**
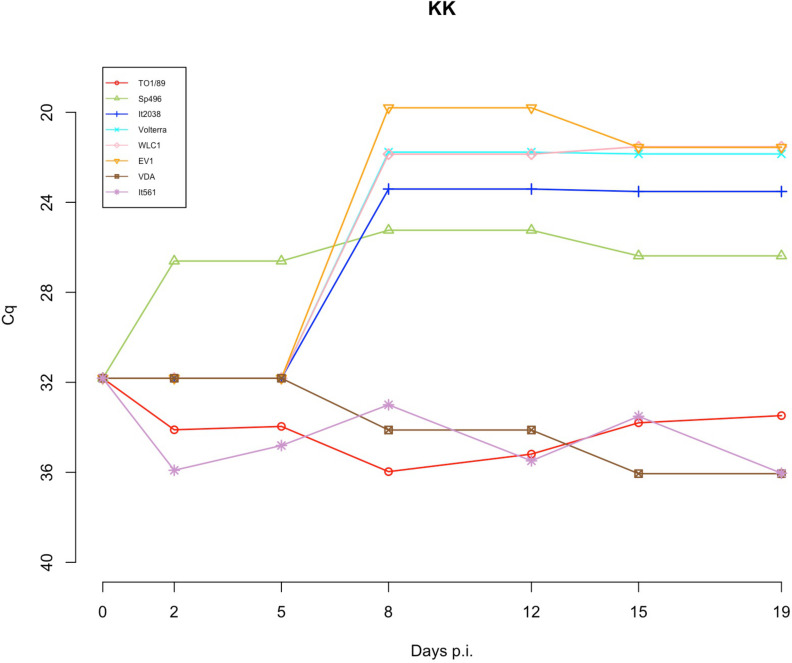
Viral replication of the eight SRLV strains in KK cell lines homozygous for SNP E35K in *TMEM154* gene. RT activity tested through the SG-PERT assay in the supernatant of cell cultures at time from T0 to T19 days p.i.

Considering the eight viral strains, two out of eight showed a statistically significant difference (Wilcoxon rank sum test *P* < 0.05) among the three cell lines. Within the genotype A, the It561 strain Cq values at time 15 p.i. showed a statistically significant difference among the results obtained for KK and EK cell lines compared with the EE cell lines (*P* = 0.0167, confidence interval [CI] 95%), while within the genotype B, only subtype B1, strain TO1/89, showed a statistically significant difference at time T8 and T15 (*P* = 0.0285, CI 95%) (Fig. 9 and 10).

**Fig 11:**
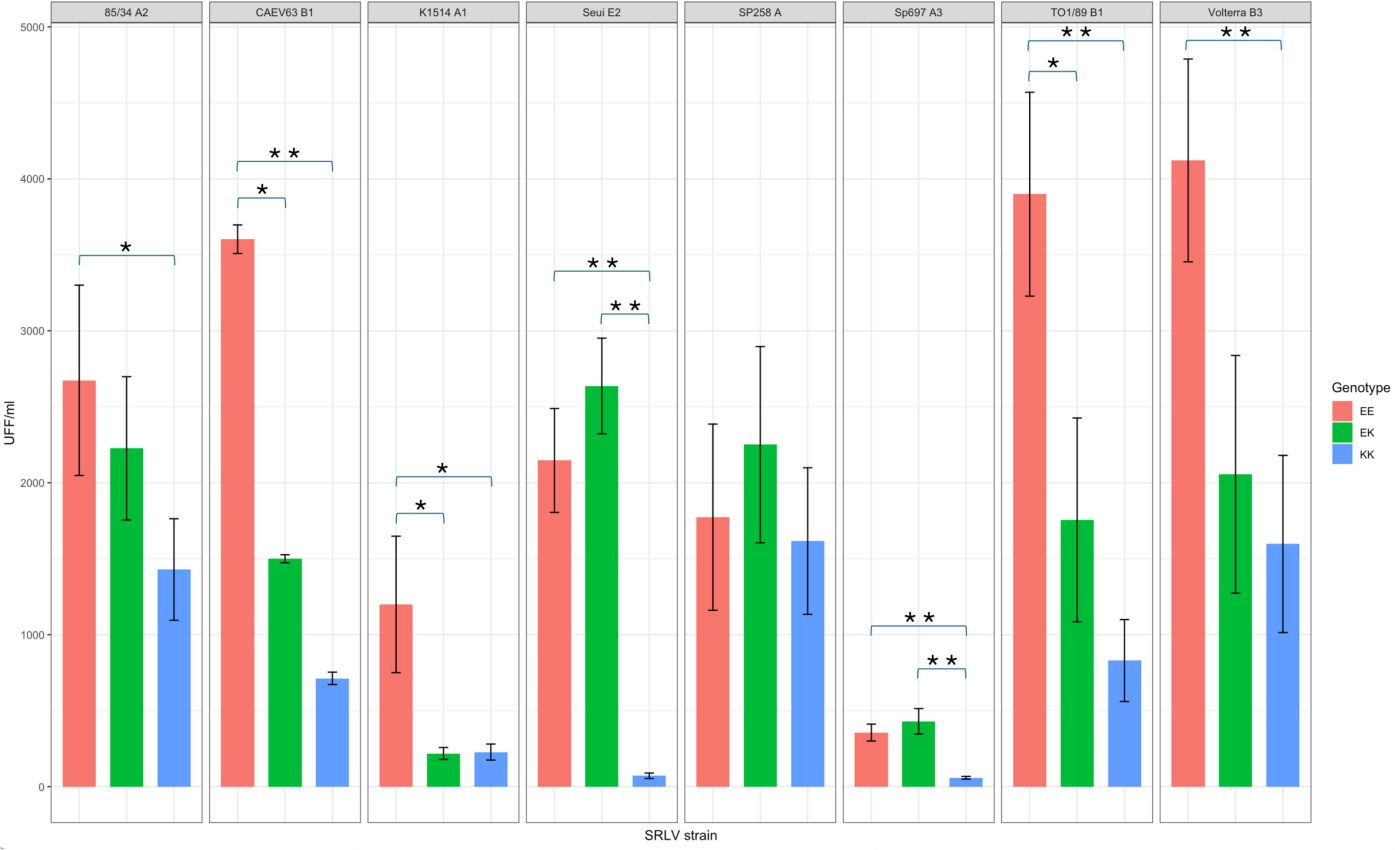
Entry assay with pseudotyped SRLV. The bar plot shows the UFF/mL obtained with eight viral pseudotypes in ovine cell lines harboring the three different *TMEM154* genotypes, KK, the carrier of the putative resistant genotypes, and EK and EE, the carrier of the putative more susceptible genotype. The bar charts represent the mean values, and the error bars show the standard deviation in five independent experiments on three cell lines for EE and KK genotypes and two for EK genotype.

To verify if the results were correlated to the differential ability of SRLV strains in entering the cells harboring a putative resistant genotype, entry assays using CAEV-AP virions pseudotyped with envelopes from different SRLV strains were performed. Among the eight viral strains investigated, all but one showed a statistically significant difference in the ability to enter the different cell lines using the Kruskal-Wallis test with Dunn’s multiple comparison test with Bonferroni correction. Figure 11 shows the results expressed in unit forming foci (UFF)/mL of the eight viral strains in five independent experiments on different cell lines for each genotype (three for EE and EK and four for KK genotypes). All but Sp258 pseudotype showed a statistically significant difference among KK and EE cell lines (*P* < 0.05), while Seui and Sp697 pseudotypes showed a statistically significant difference between EK and KK cell lines (*P* = 9.47 × 10^−8^ and 2.57 × 10^−6^, respectively, CI 95%) and K1514, CAEV63, and TO1/89 pseudotypes showed a barely significant difference between EE and EK cell lines (*P* = 0.043, 0.016 and 0.021, respectively, CI 95%). A higher number of UFF has been verified in EE cell lines compared to KK cell lines in B1 CAEV63 and TO1/89 (*P* = 0.00013 and 6.82 × 10^−5^), B3 Volterra (*P* = 0.00035) and A1 K1514 (*P* = 0.0069), A2 85/34 (*P* = 0.00175), E2 Seui (*P* = 9.47 × 10^−8^), and A3 Sp697 (*P* = 2.57 × 10^−6^) pseudotypes.

Asterisks denote statistical significance (*P < 0.05; **P < 0.001).

## DISCUSSION

SRLV infections are widespread globally and represent one of the major challenges in small ruminant production. The virus causes a lifelong infection and varied clinical manifestations for which no effective therapy or vaccine is available to date. For this reason, many countries have implemented control and eradication programs that combine management strategies with sequential testing approaches (18, 48). However, the antigenic spectrum of SRLV is not always covered by commercial tests (7, 48, 49). Therefore, over the last decade, starting from the pioneering work by Heaton and colleagues in North America (20), a number of studies have investigated the role of *TMEM154* gene polymorphisms in the resistance/susceptibility against SRLV infection in sheep populations from the Americas (22, 24, 26, 27, 50), Europe, and Asia (29–32, 51–53). The common purpose was to investigate the possibilities offered by marker-assisted selection to control SRLV infection in sheep (23).

The *TMEM154* polymorphism, located in the region of the gene encoding extracellular domain of the protein, gives rise to three different haplotypes: haplotype 1, containing lysine (K) at position 35, and haplotypes 2 and 3, containing glutamic acid (E) at position 35 (20). The resistance pattern was highlighted only in homozygous KK animals, while heterozygous EK and homozygous EE were found to have an increased risk of infection. Nonetheless, the disease’s clinical manifestations vary according to management systems, SRLV strains, animal breed, sex, age, and immune status (21, 29, 32, 52, 54).

There is a growing body of evidence on the influence of *TMEM154* on SRLV susceptibility; however, the underlying mechanisms of resistance in KK animals remain largely unexplored. Therefore, the aim of the present study was to investigate the association between the polymorphism of *TMEM154* gene and the susceptibility of sheep cell lines to SRLV infection *in vitro* with different viral subtypes as well as to unveil possible mechanisms involved.

To this end, we utilized tenovine fibroblastic cell lines derived from the previous experiment and belonging to Biellese breed seronegative animals carrying the three possible genotypes of *TMEM154* gene (KK, EK, or EE).

Although it is well established that monocytes/macrophages are the primary target cells of SRLVs, we deliberately chose to use fibroblastic cell lines in the initial phase of our study. This decision was based on both biological and technical considerations. SRLV infection is strongly influenced by macrophage polarization, with M2 macrophages being more susceptible than M1. Both the culture conditions and the virus itself can modulate macrophage differentiation, potentially altering SRLV susceptibility and confounding the interpretation of experimental results (55). This is due to the high phenotypic plasticity of macrophages, which contributes to the instability and heterogeneity of macrophage populations *in vitro*. Fibroblasts, on the other hand, are also natural targets for SRLVs (56–58) and offer several technical advantages, including greater stability in culture and higher transfection efficiency. For these reasons, we chose to generate TMEM154 mutants and perform our experiments in fibroblasts, allowing for a more consistent genetic background and a controlled cellular environment. By contrast, macrophages are notoriously difficult to transfect, and creating TMEM154 mutants in these cells while maintaining a stable genetic background would present substantial technical challenges.

Cell lines were then infected with eight SRLV strains, WLC-1 and EV-1 (A1 subtypes), It561 (A3 subtype), VDA (A8 subtype), TO1/89 (B1 subtype), It2038 and Ov496 (B2 subtype), and Volterra (B3 subtype) and monitored for 19 days for the presence of cytopathic effect and RT activity through RT-PERT Sybr Green I assay.

Early after infection, A1 subtypes WLC-1 and EV-1 showed the ability to replicate in the monolayer of every cell line, causing a strong cytopathic effect consisting of extended lysis and small syncytia. This result was confirmed by viral titration assay that outlined similar Cq values in the supernatant of every infected culture and can be explained by taking into account that the two viruses are considered highly virulent strains but also well-adapted viruses as a result of several passages in cell culture. Thus, it should be considered that wild-type A1 strains could give slightly different results.

Similarly, B2 subtypes Ov496 and It2038 were able to replicate in all cell lines regardless of the genotype. The results correlate with microscopy findings, represented by small- and medium-sized syncytia in all three cell lines infected with It2038 strain, while a minimal difference in the number and size of syncytia was observed between EE and EK versus KK cell lines infected with Ov496 strain. Similarly, for the B3 subtype, Volterra strain did not show differences since medium-/big-sized syncytia and similar RT activity have been observed between the three infected cell lines.

Conversely, in compliance with the results of the viral titration assay and cytopathic effect, A8 VDA subtype was not able to replicate in any cell line. The A8 subtype has been suggested to be a low pathogenic subtype infecting goats (4), as A4 subtype in Switzerland (59, 60), which may have circulated in flocks undetected, and this result confirms its low ability to replicate in ovine fibroblastic cells. This hypothesis is in accordance with previous studies that showed how some SRLV strains exhibit an impaired ability to infect fibroblastoid cells together with a strict tropism for macrophages, as for E1 Roccaverano strain (40, 60, 61).

Regarding the RT activity results, statistically significant differences among cell lines were recorded for two viruses.

Of particular note are the results obtained for B1 subtype. The TO1/89 strain, in fact, only replicated in cell lines homozygous or heterozygous for the wild-type allele G (E35), resulting in numerous small- and medium-sized syncytia and low Cq values. Conversely, in the cell lines homozygous for the putative resistant allele A (K35), no RT activity or cytopathic effect was recorded, except for a small syncytium in one out of 4 KK cell lines. Small differences among the cell lines tested within the same *TMEM154* genotype and infected with a particular strain could be attributed to the differential expression of other antiviral or proviral genes. Data analysis confirmed the presence of a statistically significant difference between the results obtained in homozygous KK cell lines and EK or EE cell lines in the case of TO1/89 strain, B1 subtype (*P* = 0.0285, 95% CI). The *in vitro* infection results suggest that the homozygous genotype AA (KK) is protective against SRLV infection against B1 subtype, TO1/89 strain. Lastly, regarding the It561 strain, A3 subtype, a slow-replicating pattern was observed in homozygous EE cell lines starting from T15 alongside the appearance of a limited number of small-sized syncytia. Thus, strain It561 showed a preferential ability to replicate in EE cells, being restricted in KK fibroblasts, according to RT results. Accordingly, our recent serological results indicate an association between the animal genotype for the putative protective mutation of the gene *TMEM154* and the infecting virus genotype, in three different sheep breeds reared in northern Italy (32) confirming a protective effect only for SRLV genotype A strains. Moreover, a statistically significant difference was identified in cells infected with the It561 strain, A3 subtype, between cell lines homozygous for the risk-associated allele G (EE) and the cell lines heterozygous and homozygous (EK and KK) for the putative resistant allele A (*P* = 0.0167, 95% CI). Furthermore, a cellular genotype carrying at least one copy of the A allele (K35) can confer resistance against A3 subtype, It561 strain, since infection in KK and EK cell lines was abrogated.

To further explore the host/pathogen interaction and the restriction mechanism, an entry assay using the envelope of several SRLV subtypes was performed. Considering the results obtained in the *in vitro* infection assay, cell lines encoding the three different genotypes were transfected with CAEV-AP virions pseudotyped with envelopes from SRLV strains homologous to those used in RT experiments. Namely, K1514 homologous to the Icelandic strain WLC1 (subtype A1), the 85/34 and Sp697 homologous to the It561 strain (subtype A3), and CAEV63 homologous to the TO1/89 strain (subtype B1). In addition, virions pseudotyped with Sp258 (A genotype), TO1/89 (B1 subtype), Volterra (B3 subtype), and Seui (E2 subtype) strains were tested, while we were not able to obtain a B2 subtype pseudotype.

The assay revealed statistically significant differences in viral entry efficiency between KK and EK/EE cell lines for all tested subtypes, except Sp258 (A strain), suggesting that the resistance conferred by TMEM154 is mediated by an entry blockade. Although TMEM154 function has not been revealed, domain analysis indicates the presence of TM motifs potentially locating the protein at the plasma membrane. Likely, once a certain level of entry is accomplished, replication in EK or KK cells reaches similar values, as in the case of Volterra strain.

The putative resistant lines, both in homozygosis and heterozygosis (KK and EK), in fact, showed a clear pattern of restriction, with a limited number of focus forming units in contrast to permissive cell lines for the B1 subtypes TO1/89 and CAEV63, as well as for A1 K1514 subtype. Accordingly, 85/34 A2, Seui E2, Sp697 A3, and Volterra B3 subtypes were restricted in homozygous (KK cell lines) of the putative protective allele A (K35).

These variations can be explained by considering the various virus binding sites and differential receptor recognition used by different SRLV strains as previously demonstrated for SRLV and other lentiviruses (62–64). Variation in the *TMEM154* expression levels in the different cell lines obtained could also have shaped the ability of SRLV to enter and progress.

Taken together, the results of the present research confirm the conclusions obtained in previous studies on the possibility of using *TMEM154* locus as a valid marker for genetic selection or as the opposite, a good predictive factor for greater susceptibility to the infection with certain SRLV strains (20, 22, 25, 26, 29, 51, 65). Moreover, in most of these studies, it is highlighted the importance of better investigating the role of SRLV genetic diversity in the host/pathogen interaction. To the best of our knowledge, this is the first study unveiling the *TMEM154* mechanism responsible for SRLV resistance *in vivo* by describing *in vitro* restriction patterns related to viral entry.

It should be pointed out that these results have been obtained in cell lines belonging to only one sheep breed, the Biellese one, and Ramirez et al. (52) suggested that the relation between *TMEM154* genotype and SRLV resistance/susceptibility could be limited to some breeds since they reported a positive association only in one out of four sheep breeds investigated. This finding is in accordance with other studies that reported a high number of seropositive animals in sheep with EK or KK *TMEM154* genotypes (29, 30, 32). Thus, the use of genetic selection based on *TMEM154* genotype should consider both ovine breed and SRLV circulating strain factors. In our research, a clear pattern of resistance was recorded for B1 subtype. It is noteworthy that B1 is frequently associated with the clinical form in goats, to the extent that it is the target of specific eradication programs in several countries. However, in the majority of these programs, the sheep population is not tested, despite the occurrence of subtype B2 highly associated with arthritis in sheep (66). Indeed, Ov496 strain was isolated from an arthritic goat from the Raza Aragonesa breed which displays a high proportion of KK SNP leading to the consideration of a resistant breed (23). Considering that the cross-species transmission of this subtype was demonstrated (67–69), the risk that sheep may represent a reservoir of B1 subtype in case of eradication in the goat population is plausible. The possibility of applying a genetic selection against this viral cluster in ovine species would provide a good opportunity to reduce the risk of reintroduction of B1 subtype in the more susceptible caprine species.

In conclusion, *TMEM154* E35K polymorphism seems to be confirmed as a valuable marker for the genetic selection of resistant or less susceptible animals to some SRLV subtypes or, on the other hand, a helpful predictive factor for greater susceptibility to SRLV infection. Our results suggest that this is especially true for B1 strains; however, some A and especially B2 strains escape from the restriction exerted by *TMEM154*. Different factors such as differential receptor usage of B2 strains avoiding interaction with TMEM154, hijacking of the activation cascade potentially derived from TMEM154 engagement, or the intervention of innate immune factors are plausible explanations (70). This study also validates the *ex vivo* approach as a valuable tool for investigating SRLV resistance mechanisms. Although *in vivo* confirmation is needed—such as genotyping seropositive and seronegative animals from flocks with known B1 circulation—our cell lines provide a reliable *in vitro* model for studying resistance patterns against various SRLV strains. Furthermore, it was confirmed the crucial role of the accurate detection and monitoring of circulating viral variants for the effective control of SRLV infection through marker-assisted genetic selection programs. Further studies are needed in order to better investigate other polymorphisms of *TMEM154* gene as well as other host genetic markers. These insights, together with the analysis of viral strains circulating in certain areas, could represent a valuable tool for the implementation of effective control and eradication programs for SRLV infection in ovine species. Since comprehensive consideration of sheep and goats is needed in control plans due to the SRLV ability to cross species barrier, future studies should address *TMEM154* genotypes and their significance on SRLV infection in goats.

## Data Availability

The data sets generated and/or analyzed during the current study are available from the corresponding author upon reasonable request.
